# A cross-sectional epidemiological study of tuberculosis in the province of Sidi Kacem, Morocco

**DOI:** 10.11604/pamj.2023.44.106.25958

**Published:** 2023-02-24

**Authors:** Abderrahmane Boualam, Rajaa Seghiri, Driss Touil, Naby Berrid, El Mahjoub Aouane

**Affiliations:** 1 Laboratory of Natural Resources and Sustainable Development, Department of Biology, Faculty of Sciences, Ibn-Tofail University, B.P. 133, 14000, Kenitra, Morocco,; 2 Laboratory of Advanced Materials and Process Engineering, National School of Chemistry, Ibn Tofail University, B.P 242, Kenitra, Morocco,; 3 Laboratory of Biology and Health, Department of Biology, Faculty of Sciences, Ibn Tofail University, B.P. 133, 14000, Kenitra, Morocco

**Keywords:** Pulmonary tuberculosis, extrapulmonary tuberculosis, a bacterial disease, incidence, lethality

## Abstract

**Introduction:**

according to the World Health Organization (WHO), tuberculosis (TB) is a global emergency and a leading cause of death as a world bacterial disease. This dangerous disease affects poor and vulnerable populations, mainly seniors and children. This study aimed to describe the epidemiological profile of tuberculosis; based on clinical and evolutionary socio-demographic characteristics in the province of Sidi Kacem.

**Methods:**

our study concerned the cases of tuberculosis diagnosed and treated in the center of tuberculosis and respiratory diseases of Sidi Kacem during the years 2018 and 2019. The data were collected from the medical records of tuberculosis patients.

**Results:**

we recorded 1059 tuberculosis patients, representing an average rate of 100.77 new cases per 100000 inhabitants. Males represented 64.5% (n=683). The age average was 34.94±16.73 years. The majority of patients, 68.36% (n=724), are between 15 and 44 years old. Extrapulmonary TB represented 42.12% (n=623) compared to 58.88% (n=623) pulmonary TB, 78.30% (n=487) who had positive results for bacilloscopy. A lethality percentage of 1.7% (n=18) had observed.

**Conclusion:**

tuberculosis in the province of Sidi Kacem is still claiming victims, and all sections of society exposing to this disease. Tuberculosis is more dangerous when it affects the lungs because this form is the one that contributes to infection and spread of the disease and causes more deaths. We hope that this research presented here will stimulate more strategies for adequate and specific case management of pulmonary tuberculosis and encourage treatment adherence.

## Introduction

Tuberculosis (TB) is a widespread infectious communicable disease worldwide. It can be lethal in some instances [1]. According to the Global tuberculosis report of the world health organization (WHO), the estimated incidence of TB is approximately 1490 thousand of cases death in 10 million individuals affected by TB, in 2018, up from 10 million cases with 1.6 million deaths in 2017 [2,3]. The WHO estimated that 10.4 million new cases (incidents) of tuberculosis were reported at the international level in 2015, of which 5.9 million (56%) were among men, 3.5 million (34%) among women, and 1 million (10%) among children [4]. All the countries are affected, but most cases (85%) occur in Africa (30%) and Asia (55%), whereas India and China formed together 35% of the total [2]. Lung damage is the most frequent localization (80%) of cases, which represents the main transmission source. Thus, the bacillus can reach other organs (often the pleura, lymph nodes, spine, joints, genitourinary tract, nervous system, and so on) causing extra-pulmonary tuberculosis [5]. In this sense, the annual number of Tuberculosis cases in Morocco has been estimated at 36000 in 2014. The estimated number of 28955 new cases and 1681 relapses arising in the following year has expressed as the rate per 30636 cases of all Tuberculosis cases. Therefore, the incidence reached in 2015 has identified 89 cases per 100000 inhabitants. Moreover, the number of deaths was 656 cases have died by TB, and 160 patients developed a multi-resistant TB. According to what was presented above, the overall incidence of TB decreased by 17 %. Whereas, pulmonary TB decreased by 20%, from 2000-2015. Also, a high proportion of extra-pulmonary TB (EPTB) 52% of cases was reported. It was 33% of primary tuberculosis cases compared to 48% of pulmonary TB cases [6]. Bacteriologically, the confirmed TB cases in 2015 accounted for 44% of reported cases, compared to 56% of cases diagnosed according to clinical criteria. The age distribution reveals that almost 2/3 of the cases were young adults aged between 15 and 44 years. Moreover, 60% of the recorded cases were male [6]. The geographical distribution shows that only5 regions accounted for 58% of reported tuberculosis cases, with an incidence above the national average. These regions are Casablanca, Tangier-Tetouan, Rabat-Salé-Zemmour-Zaër, Fes-Boulemane and Gharb-Chrarda-Beni-Hssen [6]. Tuberculosis causes more deaths in the province of Sidi Kacem. This study is designed to contribute to the reflection on how to reduce the high rate of lethality caused by this disease and encourage treatment adherence. This study aimed to describe the profile epidemiology of tuberculosis; based on clinical and evolutionary socio-demographic characteristics in the province of Sidi Kacem.

## Methods

**Study design and setting:** we conducted a retrospective descriptive study from January 2018 to December 2019 at the center of tuberculosis and respiratory diseases of Sidi Kacem. The province of Sidi Kacem is the predominantly rural subdivision of the Rabat-Salé-Kénitra region located in the northwest of Morocco. It takes its name from its Head-Lieutenant Sidi Kacem, it is composed of 15 communes, including 5 urban communes: Sidi Kacem, Mechra Bel Ksiri, Jorf El Melha, Had Kourt, and Dar Gueddari. The population is 692,239 (based on the 2015 census). On the axes Meknès (45 km) - Tangier (210 km) and Fez (85 km)- Rabat (120 km).

**Participants and data collection:** we included all tuberculosis patients declared at the diagnostic center for tuberculosis and respiratory diseases (DCTRD) during the study period. We have excluded the incomplete files. The tuberculosis patients' data had collected on a standardized form which included socio-demographic data (age, sex, place of residence), clinical characteristics, types of tuberculosis (pulmonary TB, extra-pulmonary TB or mixed), therapeutic evolution (cures, death, loss of sight, transfers, abound failures) after the treatment period.

**Statistical analysis:** data were collected and analyzed using SPSS software version 22.0 (Chicago, IL, USA). The qualitative variables had summarized in percentages (%). The statistical comparisons had made with the chi-square test for categorical variables. A p-value less than 0,05 was considered statistically significant.

**Ethical consideration:** to conduct this study and collect data from patient records at the tuberculosis and respiratory disease center in Sidi Kacem, we received authorization from the delegation of the Ministry of Health of the province of Sidi Kacem dated February 7^th^, 2019 number 223B.R.H.No verbal or written informed consent was obtained from the patients as this was a retrospective study. However, the patients' identities were kept confidential.

## Results

**Incidence of TB and socio-demographic characteristics of patients:** in the level of the epidemiological and clinical characteristic, the results showed that the incidence rate was lower with 69 cases per 100,000 inhabitants in 2011 and increased to a higher rate in 2018 with 103 cases per 100,000 inhabitants, then a slight decrease in 2019 (Table 1). According to socio-demographic parameters, the results show that both genders were affected by a male predominance. Indeed, it found that 682 males compared to 377 females with a male-female ratio of 1.8 in favor of males. Also, the table presents the repartition of tuberculosis by age group, and it shows that the age range from 15 and 44 years old is the most affected with 68.36%. At the same time, this disease is rarely detected in children under 15 years old. However, it is significantly detectable in those over 65 years. Besides, 54% of patients live in rural areas, against 46 %in urban areas (Table 2). The majority of patients are from the Chrarda circle with 314 cases which represent 29.7% of TB patients. The two Ouargha circles and Gharb Bni-Malek have a very close staff of 234 tuberculosis for Ouargha and 213 for GharbBni-Malek, followed by the Baht circle with a staff 129 infected patients. While the Tilal-lgharb circle has the lowest effect with 100 cases of TB (Table 3).

**Table 1:** evolution in the incidence of all forms of tuberculosis per 100,000 inhabitants between 2011-2019 in Sidi Kacem

Years	ni	Incidence/10^5^
2011	334	69.43
2012	379	77.89
2013	322	95.58
2014	426	85.93
2015	414	82.81
2016	500	98.82
2017	494	98.24
2018	595	103.31
2019	518	98.41

**Table 2:** characteristics of tuberculosis cases

Variables	Categories		percent
**sex**	Man		35.5%
	woman		64.5%
**Age range**	0-14 years		2.64%
	15-24 years		25.40%
	25-34 years		34.37%
	35-44 years		8.59%
	45-54 years		11.52%
	55-64 years		6.99%
	More than 65		10.48%
**Environment**	Urban		46%
	rural		54%

**Table 3:** distribution by residence

Circle	ni	percent
Baht	129	12.2%
Chrarda	314	29.7%
Gharb bnimalek	213	20.1%
Tilallgharb	100	9.4%
Ouargha	234	22.1%
Out of Province	69	6.5%

**Clinical presentation:** when we turn to the distribution of all forms of TB cases by location, the results show that on one hand, 446 isolated extra-pulmonary TB cases were observed. Thus, an incidence of 42.12% of cases. On the other hand, two cases of lung and extra-pulmonary combination disease were observed. Thus, an incidence of 0.19% of cases. Consequently, pulmonary tuberculosis accounted for 57.69% of TB. Based on the results obtained, the extra-pulmonary form pleural diseases predominate with 21.06% of all tuberculosis forms cases. Followed by ganglionic and peritoneal diseases, which account for 8.69% and 2.64% of all tuberculosis forms cases (Table 4).

**Table 4:** distribution based on location

Forms of tuberculosis	ni	Percent(%)
**Pleural**	223	21.06
**Node**	92	8.69
**Peritoneal**	28	2.64
**Osteo-articular**	23	2.17
**Miliary**	15	1.42
**Genito-urinary**	8	0.76
**Pericarditis**	5	0.47
**Skin**	5	0.47
**Meningitis**	5	0.47
**Others**	42	3.97
**Pulmonary**	613	57.88
**Total**	1059	100

**Treatment results:** according to therapeutic outcomes distribution, we have been finding out that the evolution of the disease is variable. The cured patients showed a percentage of 77.34% (n=819) of cases. Both patients and abundance do not exceed 1% (n=2) of cases. The patients missed represented a significant percentage of 13.31% (n=141). The average fatality rate was 1.6% (n=18) for eight patients under 44 years old; nine patients aged 45 to 64 years, and one tuberculosis patient over 64 years old. We recorded more deaths in the group of patients with pulmonary tuberculosis, i.e. 78% (n=15) than those with extra-pulmonary tuberculosis (Table 5).

**Table 5:** distribution according to therapeutic outcomes

Evolution	Total		Extra-pulmonary TB		Smear-negative PTB		smear-positive PTB	
	n	%	n	%	n	%	n	%
Healing	819	77.34	366	4.56	104	9.82	349	32.95
Death	18	1.7	3	0.28	4	0.38	11	1.04
Loss of sight	141	13.3	49	4.63	21	1.98	71	6.70
Transfers	74	6.99	28	2.64	4	0.38	42	3.97
Failures	5	0.47	0	0	0	0	5	0.47
Abondons	2	0,19	0	0	0	0	2	0.19
Total	1059	100	446	42.11	133	12.56	480	45.33
		Extrapulmonary TB			Pulmonary TB			Pvalue
Death		3			15			0,027*
Others(survival)		443			598			

Chi Square = 4.86, DF = 1 and p = 0.027 ; TB: tuberculosis PTB: pulmonary tuberculosis

## Discussion

Tuberculosis causes more deaths in the province of Sidi Kacem. This study was designed to contribute to a reflection on how to reduce the high rate of lethality caused by this disease and encourage treatment adherence. This study aimed to describe the profile epidemiology of tuberculosis. Based on clinical and evolutionary socio-demographic characteristics in the province of Sidi Kacem. We recorded 1059 tuberculosis patients, males representing 64.5% (n=683). The majority of patients, 68.36% (n=724), were between 15 and 44 years old. Extra-pulmonary TB represented 42.12% (n=623) compared to 58.88% (n=623) pulmonary TB, 78.30% (n=487) who had positive results of bacilloscopy. A percentage of 1.7% (n=18) lethality was observed. The study has found that both genders were affected by a male predominance. The gender ratio varied between 1.99 and 2.28 in favor of men. This result by Bercion R *et al*. in Yaoundé reports a gender ratio of 1.5 for men [7]. Indeed, men are more vulnerable to infection than women because the latter are less productive economically and have difficulty accessing healthcare services. This probability gets higher, especially in developing countries, due to traditions in Sidi Kacem Province. Thus, men are at greater risk of acquiring tuberculosis due to its presence in overcrowded areas such as markets and farms. In addition, men are more susceptible to drug and smoking addiction than women.

Tuberculosis is a disease that affects individuals at any age in their lives. In this study, the age group from 15 to 44 was the most affected at 76%, while those aged between 45 and 65 represented only 16%. These results are comparable to those found in the Maghreb countries, where the age group between 20 and 45 years is the most affected by the disease by 70%, the most economically productive group causing economic and social disruption. It represents more than half of the cases reported in developing countries, and this is the case in Algeria, Morocco, and Tunisia [8]. In developing countries, the disease primarily affects people between the age of 15 and 45 in 75% of cases. In parallel, it mainly affects people over 70 years old in developed countries. In France, the most affected people are over 80 years old [9]. Pulmonary tuberculosis accounted for 57.88% of the tuberculosis cases recorded during the study period. This value is lower than those reported in the previous studies, in which 56.4% and 89.6% of cases had localized at the pulmonary level found in a nearby province Larach in Morocco, and Bercion in Cameroun, respectively [7-10]. In parallel, this value was lower than those reported in 68%, 78%, and 89.6% of pulmonary forms cases found in other African countries, Tunisia, the Republic of Ivory Coast, and Cameroun, respectively [7,11,12].

In our study, isolated extra-pulmonary TB was observed in 442 cases, representing an incidence of 41.8% of cases. Four (4) patients had a primary infection. The study by Kruijshaar ME *et al*. (2009) in England showed that more than 40% of reported TB cases are extra-pulmonary TB, while the percentage of extra-pulmonary TB varies from 7% to 25% in France [13,14]. The preponderance of pulmonary localization is a part of the normal distribution of tuberculosis, which demonstrates the value of early detection and adequate treatment of cases [15]. Pleural involvement (21.06%) is the most frequent clinical form of extra-lung tuberculosis, as also found by Cherif *et al*. 2014 in their studies [12]. In our study, the lethality rate of tuberculosis was estimated at 1.7%. And this rate is lower than those found in the study of D. Che and Antoine 2011; Bakhat 2010, which are respectively 11% and 14% (8,9). The highest mortality was found in patients with pulmonary tuberculosis at 83.33% (Chi-Square = 4.86; DF = 1, and p = 0.027). In our observation, this clinical form remains the most lethal. The risk of death is higher when suffering from pulmonary tuberculosis is confirmed by microscopy than other forms of tuberculosis. This study on tuberculosis is the first in the province of Sidi-Kacem, which can contribute at the local level by targeting the segment the most exposed to this disease, and the most affected areas to intervene appropriately and effectively. We encountered problems with the lack of information in the patients' files, which prevented us from knowing their therapeutic future.

## Conclusion

Despite the efforts of all actors in the health sector, tuberculosis in the province of Sidi Kacem still claims many victims because all sections of society are being exposed to this disease. Tuberculosis is more dangerous when it affects the lungs because this form is the one that contributes to infection and the spread of the disease and causes more deaths compared with extra-pulmonary tuberculosis. We hope that the research presented here will stimulate more strategies for diagnosis, adequate and specific management of cases of pulmonary tuberculosis, and encouraging treatment adherence.
